# Screening of Swiss Pig Herds for Hepatitis E Virus: A Pilot Study

**DOI:** 10.3390/ani11113050

**Published:** 2021-10-25

**Authors:** Julia Lienhard, Isabelle Vonlanthen-Specker, Xaver Sidler, Claudia Bachofen

**Affiliations:** 1 Institute of Virology, Vetsuisse Faculty, University of Zurich, 8057 Zurich, Switzerland; lienhard.julia@gmail.com (J.L.); isabelle.specker@outlook.com (I.V.-S.); 2 Division of Swine Medicine, Department of Farm Animals, Vetsuisse Faculty, University of Zurich, 8057 Zurich, Switzerland; xsidler@vetclinics.uzh.ch

**Keywords:** hepatitis E virus, pig, screening, genotyping, Switzerland

## Abstract

**Simple Summary:**

Hepatitis E is a viral disease found in humans worldwide. The hepatitis E virus (HEV) is also found in various animals, with pigs as the main reservoir. A transmission of the zoonotic genotype 3 to humans is possible via direct contact with infected animals or through the consumption of raw or undercooked meat. A good knowledge of the genetic diversity of the virus in pigs is needed to prevent future transmissions by tracing chains of infections. Thus, this study aimed at developing a practical and easy to use screening tool for pig herds and gaining first experiences with it. Sock swabs turned out to be the method of choice and were used to test 138 Swiss pig herds for HEV. In positive cases the virus was further characterized. Of the 138 farms tested, 81 were HEV positive (58.8%) and most viral sequences belonged to a distinct cluster within subtype 3h that was also commonly found in Swiss patients infected with HEV. These data showed that sock swabs are useful for HEV screening in pig herds, that HEV is frequent in Swiss pig herds, and that the same subtype of virus is found in Swiss pig farms and human patients.

**Abstract:**

Hepatitis E virus (HEV) is an important cause of acute hepatitis in humans worldwide. In industrialised countries, most infections are caused by the zoonotic genotype 3. The main reservoir was found in pigs, with fattening pigs as the main shedders. The aim of this study was to establish a screening tool to detect HEV in pig farms. HEV-positive samples were sequenced using Sanger sequencing. First, different sample materials, including floor swabs, slurry, dust swabs and faeces were tested for HEV. Floor swabs turned out to give the best results and, in the form of sock swabs, were used for the screening of Swiss pig herds. A total of 138 pig farms were tested, with a focus on fattening pigs. Overall, 81 farms (58.8%) were HEV positive. Most sequences belonged to subtype 3h, in which they formed a specific cluster (Swiss cluster). In addition, subtype 3l and two unassigned sequences were detected. As a conclusion, sock swabs were found to be a helpful tool to screen pig herds for HEV and establish a sequence collection that may enable molecular epidemiology and support outbreak investigation and prevention.

## 1. Introduction

Hepatitis E virus (HEV) is a single-stranded, positive-sense RNA virus that belongs to the species *Orthohepevirus A* within the family *Hepeviridae*. It is a small virus with a genome length of about 7.2 kb containing three open reading frames (ORFs) [1]. HEV is considered quasi-enveloped, because it is non-enveloped when shed in faeces, but contains a lipid membrane in the blood circulation and in cell culture [2,3].

In developed countries, most infections are caused by the zoonotic genotype 3. The main reservoir of genotype 3 was found in domestic pigs. However, the virus was also detected in other animals, such as wild boar, deer, or rabbits [4]. Transmission to humans can occur by ingestion of raw or undercooked meat, but also by direct contact with infected animals [5]. Due to increasing numbers of HEV infections in humans in Europe, it was discussed whether HEV is an emerging disease in industrialised countries [6]. However, several countries, including Switzerland, have reported a decrease in the seroprevalence of HEV in humans over the last few years [7,8,9,10]. Thus, the previous increase in infections was more likely a result of increased awareness of HEV and improvement of diagnostic tools. Regardless of the overall tendency of HEV cases, a recent increase in HEV infections in Switzerland in early 2021 highlights the threat HEV still poses (https://www.bag.admin.ch/bag/de/home/zahlen-und-statistiken/zahlen-zu-infektionskrankheiten.exturl.html; accessed on 6 September 2021). To determine sources of infections and transmission pathways, a monitoring system in pig herds could be helpful to gain more information on possible reasons for outbreaks in humans.

In pigs, infections are caused by faecal–oral transmission, either through direct contact with infected pigs or via a contaminated environment [11,12]. Different environmental samples from pig farms, such as pen floor faecal samples or manure, were found to be HEV positive [13,14]. Piglets are protected by maternal antibodies, but shortly after the decline of the passive immunity at the age of 2–3 months, most pigs get infected [15]. While viremia only lasts 1–3 weeks, the virus can be shed for up to 7 weeks in faeces [16]. Several studies found pigs at the age of 3–4 months to be the main shedders [17]. Natural as well as experimental infections are asymptomatic in pigs [15,16]. A high HEV prevalence in pigs was found in different countries [18,19,20,21]. The seroprevalence of pigs at slaughter age in Switzerland was similarly high, with approximately 60% both on an individual and a farm level [22,23]. However, there are no data available about the viral prevalence of HEV in pig farms in Switzerland.

Hepatitis E viruses have a high genetic diversity, with several existing subtypes within genotype 3. However, no uniform subtype demarcation criteria are available, and the number of proposed new subtypes is steadily increasing [24,25]. Recent literature suggests a fixed p-distance cut-off for full-length sequences resulting in 11 subtypes and a couple of unassigned sequences within genotype 3 [26]. In Switzerland, a new subtype was suggested in 2017, when a full-length genome sequence with less than 88% homology to any known strains was isolated from a Swiss patient with a chronic Hepatitis E virus infection [27]. Subsequently, three more full-length genome sequences, isolated from a Swiss patient and a raw pork sausage and from a Swiss pig liver, were described [28,29]. Those sequences shared 95% identity with the first Swiss sequence described by Wang et al. [27], and the existence of a Swiss-specific subtype, referred to as 3s(p), was proposed. However, using the p-distance cut-off of 0.093, these 3s(p) sequences belong to subtype 3h, which was mainly found in France and a single “outlier” sequence in Mongolia [26,30]. In Swiss patients infected with HEV, this subtype 3s(p), or 3h, was found to be the most common subtype [31]. On the other hand, in Swiss pigs there are little data available about the genetic diversity of HEV. However, a large, sequence database, such as the ones existing for bovine viral diarrhea virus (BVDV) in Switzerland and Scotland [32,33] would be helpful for molecular epidemiology generally and molecular tracing specifically. Furthermore, such a database would provide valuable information on the origin of infections and the pathway of transmission in pigs. This knowledge is needed to implement measures that can prevent future transmissions. The importance of a One Health network and sequence repository for HEV has been recognised already in 2015 by the European Centre for Disease Prevention and Control (ECDC) and has led to the formation of a European expert group and exchange platform called HEVnet, which includes an international database containing HEV sequences and metadata from different sources [34].

The aim of this study was to establish a screening tool for HEV in pig herds that is practical, non-invasive, cost-efficient, and suitable for sequencing. This screening tool was then used in a pilot field-study to gain first information on the occurrence of HEV and its genetic diversity in Swiss pig herds.

## 2. Materials and Methods

### 2.1. Samples

#### 2.1.1. Evaluation of Different Sample Types

Different sample types were tested for their suitability to screen pig herds for HEV. These included individual faecal samples and three different environmental samples, namely floor swabs, dust swabs and slurry samples. These archived samples were collected in 2017 and 2018 for a dissertation on antibiotic resistance at the Division of Swine Medicine of the University of Zurich [35]. Overall, samples from 22 farms were available for our study, including 14 fattening farms, 4 farrow and rearing farms, 3 farrow-to-finish farms and one rearing and finishing farm. The samples were taken at two different time points, once when the pigs were between 2 and 12 weeks old and the second time from empty and cleaned pens after the pigs were moved to the slaughterhouse. On every farm, an approximate surface size of 0.06 sqm of unclean floors and walls was wiped manually with a gauze wipe moistened with 0.85% saline solution. Additionally, a dust sample was collected from different horizontal surfaces (e.g., windowsills, feed or water pipelines and lids of piglet nests) near the pens, also with a moistened gauze wipe. From the slurry pit samples at different depths and with different consistency were mixed in a bucket. A homogenous sample of approximately 300 to 500 mL was taken and stored at −20 °C. The floor and dust swabs were taken from the same pens as the individual faecal samples. However, not all pigs within one pen were sampled (on average 11 out of 30 animals per pen were sampled, with a range of 2–23 tested animals per pen; Table S1). Furthermore, the slurry samples were taken from slurry pits, which usually contained sample material from several different pens. We first tested the environmental samples from all farms, but HEV-positive samples were only found in fattening farms and rearing and finishing farms. Therefore, only these two farm types (independent of the result for the environmental samples) were further analysed using the individual faecal samples.

#### 2.1.2. Sock Swab Screening

Among the environmental samples, pig pen floor samples were shown to represent the actual HEV status best and were therefore chosen for the subsequent screening pilot study. However, instead of manually wiping the floor, elastic sock swabs that can be affixed to the boots were chosen (for a picture, see Figure S1). The sock swabs were collected by various veterinarians, but primarily by members of the Swiss Pig Health Service (SPHS). We prepared test kits for the veterinarians, containing several sock swabs (one to be used per biosafety unit), slider freezer bags, padded envelopes for the prepaid return shipment, instructions for the sample collection and a questionnaire. These kits were distributed by the SPHS to their members in all regions of Switzerland. For the sampling, about 15 cm of a stockinette (SAMA Frottee Stretch Polsterschlauch, 8 cm × 10 m, reference number 380613, Smedico AG, St. Gallen, Switzerland) was used. The veterinarians were asked to moisten the sock swab with tap water and put it over one boot with the fleecy side out before walking through all pens of one unit. To prevent the spread of diseases, pens with sick animals were to be sampled last or not sampled at all. One sock swab was either used for the whole farm (if all animals were kept in the same premises) or different swabs were used for each age group on a farm. Then, each swab was put in a separate bag, sealed and returned by post. The questionnaire was filled in by the veterinarians who collected the samples and contained information on the name of the farmer, the date of the sample collection, the postal code (referring to a municipality) of the farm, the production type and size of the farm, the average age of the tested animals, any clinical signs of the tested animals and prescribed treatments at the time of sampling. The sock swabs were collected on the veterinarian’s regular farm visits between August 2020 and June 2021. Overall, 163 samples from 138 different farms were collected. Four farms that were sampled in October 2020 were sampled again in May 2021. Furthermore, on 6 farms separate samples from different age groups were collected. A farm was regarded positive if any of the samples were positive. If multiple samples from the same age group were collected the results were summarised as described above and counted as one sample per farm. Thus, overall, 153 samples were included in the analysis.

Upon arrival, the sock swabs were stored at −20 °C until testing and the data from the questionnaire were anonymised and transferred to an excel sheet. For the analysis, different age categories were used. The first group included weanlings between 4 and 10 weeks. The second group consisted of fattening pigs between 10 weeks and 6 months. The third group included pigs older than 6 months. The last group contained mixed samples from different age categories, for example samples from sows and their piglets or samples that included several age categories (Table S2).

### 2.2. RNA Extraction

#### 2.2.1. Homogenisation of Floor and Dust Swabs

The floor swabs and the dust swabs were squeezed by hand to gain the liquid. About 500 µL of the resulting liquid was pipetted in a 2 mL safe lock Eppendorf tube. The tubes were vortexed for at least 15 s and then centrifuged at 16,000× *g* for one minute. The resulting supernatant was used in the following RNA extraction.

#### 2.2.2. Homogenisation of Slurry Samples

From the slurry samples, 100 mg was weighed in a 2 mL safe lock tube or, if the sample was sufficiently liquid, 100 µL was pipetted to the tube. Then, the samples were diluted 1:10 with PBS and homogenised in the TissueLyser II (Qiagen, Hilden, Germany) for 1 min at 20 Hz before being centrifuged for 5 min at 16,000× *g*. The resulting supernatant was used for RNA extraction.

#### 2.2.3. Homogenisation of Individual Faecal Samples

For the homogenisation of the individual faecal samples, 100 mg faeces were weighed in a 2 mL Eppendorf tube and 1000 µL PBS was added. Subsequently, the samples were homogenised in the TissueLyser II (Qiagen, Hilden Germany) for 1 min with a frequency of 20 Hz and then centrifuged 5 min at 16,000× *g*. The resulting supernatant was used for RNA extraction.

#### 2.2.4. Homogenisation of Sock Swabs

All sock swabs were squeezed manually inside the plastic bag to retrieve any liquid. If swabs were too dry, 4–8 mL PBS were added, and the swabs were kneaded and soaked for a couple of minutes. The moistened swabs were added to a 50 mL conical tube partially filled with glass beads (5 mm, soda lime glass, Bohemia Cristal Handelsgesellschaft mbH, Selb, Germany). The tubes were then centrifuged for 3 min at 2000× *g* whereupon the swab remained above the glass beads and could be removed, while the fluid collected in the conus of the tube could be pipetted into a fresh tube. Of the retrieved fluid, 500 µL was added to a 2 mL tube. All samples were homogenised for 1 min at 20 Hz in the TissueLyser II (Qiagen, Hilden, Germany) and centrifuged for 1 min at 16,000× *g*. The supernatant was used in the subsequent RNA extraction.

#### 2.2.5. RNA Mini Kit

The QIAamp Viral RNA Mini Kit (Qiagen, Hilden, Germany) was used according to the manufacturer’s manual with a sample volume of 140 µL and the following modifications: no carrier RNA was used and instead of 560 µL only 555 µL of AVL buffer was added, since 5 µL of Quanti Nova Internal Control (IC) RNA diluted 1:10 with QuantiTect Nucleic Acid Dilution Buffer (QuantiNova Pathogen +IC Kit, Qiagen, Germany) was added as an extraction control. For the elution step, only 50 µL instead of 60 µL of AVE buffer was added. For each extraction, an eluate control was included. The RNA was stored at −20 °C until further processing.

### 2.3. RT-qPCR

The protocol used for the broadly reactive real-time RT-PCR was established by Jothikumar et al. [36], with the adaptation of using an MGB-modified probe as described by Garson et al. [37]. The RT-qPCR was carried out using the QuantiNova Pathogen +IC Kit (Qiagen, Hilden, Germany) according to the manufacturer’s manual, using 2 µL RNA in a final reaction volume of 10 µL. The QuantiNova IC Probe Assay, which was already added in a 1:10 dilution during the RNA extraction, was used as an extraction control. The cycling conditions were set according to the recommendations of the QuantiNova kit and reactions were run on a QuantStudio™ 7 Flex Real-Time PCR System (Applied Biosystems™, Thermo Fisher Scientific, Waltham, MA, USA).

For the first 100 reactions, all positive results were confirmed by a second RNA extraction and RT-qPCR test. However, since all results were confirmed and the Ct values showed only minor variation, further samples were only tested once. From samples tested twice, only the first Ct value was included in the data analysis.

Samples with a high IC Ct value, indicating presence of inhibitors, were repeated with a 1:10 and 1:30 dilution of the RNA. However, in most cases, this did not improve the Ct values, therefore only the first Ct value was included in the analysis. However, if a sample was negative in the first and positive in the second RT-qPCR, the sample was considered positive, and the second Ct value was included in the analysis.

### 2.4. Typing PCR

A nested RT-PCR targeting a partial ORF2 sequence with a final length of 493 nt, which was established by Boxman et al. [38], was used for HEV genotyping. In three farms, the sequencing suitability of floor swabs, dust swabs, slurry and individual faecal samples was compared. In addition, all positive sock swabs were submitted to the typing PCR.

The cDNA was synthesised using the RevertAid H Minus First Strand cDNA Synthesis Kit (Thermo Fisher Scientific, Waltham, MA, USA) according to the manufacturer’s instructions using 11 µL of RNA. The following first and second PCR were both carried out using the HotStarTaq DNA Polymerase (Qiagen, Hilden, Germany) according to the manufacturer’s manual. For the first PCR, 5 µL of cDNA was used in a final reaction volume of 25 µL and 35 cycles were run. One microlitre of the resulting product was used in the second PCR with a total reaction volume of 50 µL and 35 cycles. Afterwards, 5 µL of the final PCR product were run on a 1.5% agarose gel with GelRed^®^ (GelRed^®^ Nucleic Acid Gel Stain, Biotium, Fremont, CA, USA) staining. PCR products showing only one band with the expected size of 566 bp were purified using the QIAquick PCR Purification Kit (Qiagen, Hilden, Germany) according to the manufacturer’s instructions with an elution volume of 30 µL. If a PCR product showed several bands, the remaining 45 µL were loaded and run on a 1.5% agarose gel, and the correct band was excised and purified using the QIAquick Gel Extraction Kit (Qiagen, Hilden, Germany) according to the manual with an elution volume of 30 µL. Finally, the DNA concentration was determined on the NanoDrop^TM^ One^C^ (Thermo Fisher Scientific, Waltham, MA, USA), and samples were sent to Microsynth (Balgach, Switzerland) for bi-directional Sanger sequencing.

The software BioEdit 7.2 was used to align the forward and reverse sequences and remove the primer sequences, resulting in consensus sequences of 493 nucleotides. To resolve ambiguities, some sequences were also analysed with the software SeqMan Pro 17 (Lasergene, DNAStar Inc., Madison, WI, USA). The sequences were submitted to the online Hepatitis E Virus Genotyping Tool (Hepatitis E Virus Genotyping Tool (https://www.rivm.nl/mpf/typingtool/hev/, accessed on several dates between March 2019 and September 2021)) to assign them to a genotype and subtype [34].

### 2.5. NGS

To analyse whether sock swabs are also suitable for next-generation sequencing (NGS), which could provide additional sequence information, NGS was carried out with two sock swabs with a Ct value of 31.0 and 31.8, respectively. Both samples were prepared with three different methods to find out which one is most suitable for full-length sequencing of HEV.

For all three methods, 500 µL of the sock swab fluid was homogenised for 1 min at 20 Hz in the TissueLyser II (Qiagen, Hilden, Germany) and centrifuged for 5 min at 16,000 × *g*. For the first method, the two samples were prepared following our in-house protocol established by Kubacki et al. [39], with the exception of using only 50 µL of nuclease-free water during the RNA extraction. For the second method, the same protocol was used but for inactivation of the nucleases beta mercaptoethanol (BME) was replaced by 2 M of the less hazardous dithiothreitol (DTT; 20 µL DTT per 1 mL AVL buffer). For the third method, sample preparation was performed as for the other two methods, including filtration and nuclease treatment, but TRIzol (Invitrogen™ TRIzol™ LS Reagent, Thermo Fisher Scientific, Waltham, MA, USA) was used to extract the RNA instead of the viral RNA mini kit. For the TRIzol extraction, 150 µL of the nuclease-treated sample was topped up to 250 µL with nuclease-free water and 750 µL of the TRIzol reagent was added. Then, the extraction was performed according to the manufacturer’s instructions, and the RNA pellet resuspended in 50 µL of nuclease-free water. After amplification, samples were diluted with EB buffer to a final concentration of 3 ng. The DNA was fragmented to 500 bp using the E220 Focused-ultrasonicator (Covaris, Woburn, MA, USA). Libraries were prepared with the NEBNext^®^ Ultra™ II DNA Library Prep Kit for Illumina^®^ (New England Biolabs, Ipswich, MA, USA) according to its manual. Sequencing was performed at the Functional Genomics Center Zurich (FGCZ) using the NovaSeq 6000 (Illumina, San Diego, CA, USA) with a read length of 2 × 150 bp and paired-end sequencing. As a quality control, Phi X Control v3 Library (Illumina, San Diego, CA, USA) was used.

The SeqMan NGen 17 software (Lasergene, DNAStar Inc., Madison, WI, USA) was used to align the contigs to a database containing 739 (near) full-length sequences from genus *Orthohepevirus* received from NCBI GenBank and to the best matching HEV reference sequence. A consensus sequence was then created, using the SeqMan Pro 17 software (Lasergene, DNAStar Inc., Madison, WI, USA).

### 2.6. Phylogenetic Analysis

Phylogenetic analysis was carried out using the MEGA X software [40]. For both phylogenetic trees, a maximum likelihood tree with 1000 bootstraps was constructed. The MEGA X software was used to calculate the best fitting model. For the tree with the partial ORF2 sequences the Tamura-Nei model with a Gamma distribution with invariant sites was used. For the phylogenetic tree with the full-length sequences, the General Time Reversible model with a discrete Gamma distribution and invariant sites was used. Reference sequences received from the NCBI database were included and labelled with their accession number and their subtype according to Smith et al. [30] and Nicot et al. [26]. Reference sequences from Switzerland, formerly belonging to subtype 3s(p), are labelled with “3h s” for their subtype. All sequences from this study that were included in the phylogenetic tree were uploaded to the NCBI GenBank with the accession numbers MZ923557–MZ923643.

Two sequences that could not be assigned by the typing tool and that also did not cluster with a reference sequence in the phylogenetic tree were further analysed with the Sequence Demarcation Tool Version 1.2 (SDTv1.2) [41]. This software was used to determine the pairwise identity between the two unassigned sequences and all officially assigned full-length references of the three most closely related subtypes (3a, 3b and 3k) [26]. Furthermore, pairwise identities between the different sample types from the same farm were calculated with this software.

### 2.7. Statistical Analysis

To compare the mean and median pairwise identities of the two unassigned sequences with the members of the most closely related subtypes, statistical analysis was carried out using the program NCSS 10 statistical software (NCSS LLC, East Kaysville, UT, USA). First, Kruskal–Wallis one-way ANOVA was used to compare the medians and distributions of the pairwise identities of the three different subtypes 3a, 3b and 3k. Then, the means and medians of the two unassigned sequences and 3a and 3b references were compared pairwise using a T-Test and a Wilcoxon Rank-Sum Test, respectively. Subtype 3k was not further included in the analysis due to the small number of reference sequences in that subtype and the lower pairwise identity values compared to subtypes 3a and 3b. The *p*-value for the statistical significance was set at 0.05.

## 3. Results

### 3.1. RT-qPCR

#### 3.1.1. Evaluation of Different Sample Types

Out of the 22 tested farms, 12 farms (54.5%) had at least one positive environmental sample. Of the 15 farms at which all different sample types were tested, 14 farms had at least one HEV-positive sample (Table 1, Table S1). Positive samples were found on 13 fattening farms and on one rearing and finishing farm. Only one farm from which all different sample types were tested was negative. The sample type positive most often was the slurry sample (12 positive farms), however, in contrast to the other sample types, slurry was usually collected from several pens. Individual faecal samples were positive on 11 farms. The dust swabs had the lowest positive rate (6 positive farms), followed by the floor swabs (10 positive farms). Only on six farms were all the different sample types positive. On one farm there were no positive individual faecal samples, despite the presence of positive environmental samples, which can be explained by the fact that not all pigs within one pen were sampled. However, on two farms there were no positive environmental samples, even though there were some HEV-positive individual faecal samples. The mean Ct values of the different sample types were similarly high (Table S1).

**Figure 1:**
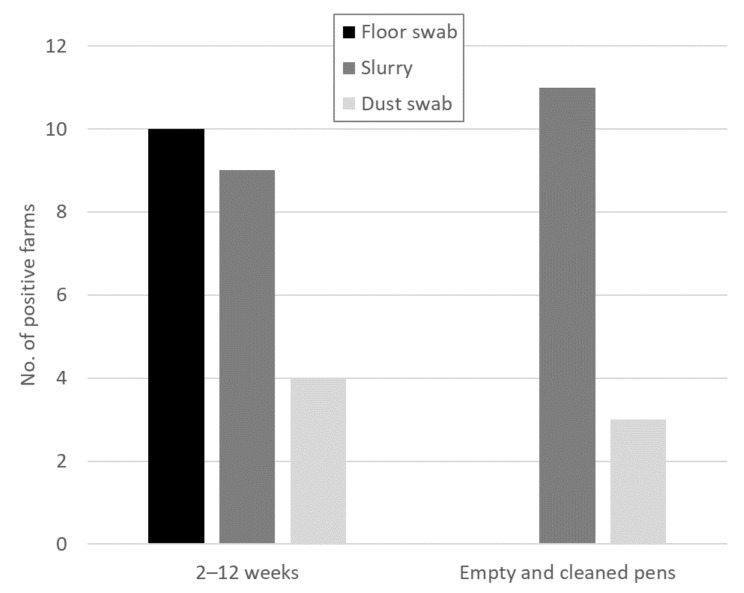
Summary of the results from the different sample types from 14 farms with HEV-positive samples at different time points. Samples were taken from farms with pigs aged 2–12 weeks and from empty and cleaned pens at the end of the fattening phase.

The floor swabs were only HEV-positive while the pigs were still in the pens. After cleaning, all floor swabs were negative (Figure 1). The slurry samples, on the other hand, were more often positive after cleaning. On eight farms, the slurry samples were positive before and after cleaning. While there was one farm that only had a positive slurry sample before cleaning, there were three farms that only had a positive slurry sample after cleaning. For the dust swabs there were positive samples on farms before (four samples) and after (three samples) cleaning, however, only on one farm was it positive at both time points.

Overall, the floor swabs and slurry samples delivered more representative results than the dust swabs. However, since more floor swabs were HEV-positive while the pigs were still on the farm, the floor swabs are a better indicator of the current HEV status on a farm than the slurry samples. Thus, sock swabs were chosen for the further screening of pig herds.

#### 3.1.2. Sock Swab Screening

**Figure 2:**
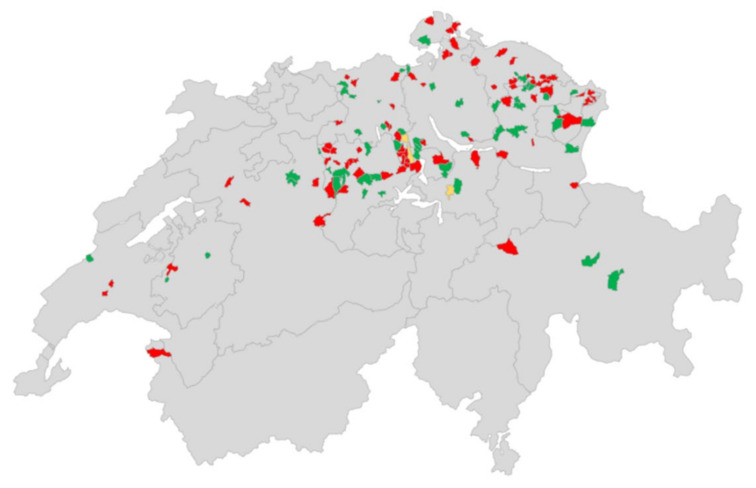
Distribution of the farms from the sock swab screening. The postcodes (from a municipality) in which sampled farms were located are marked with colour. Red colour marks postcodes with one or more HEV-positive farms; green marks postcodes with one or more HEV negative farms and yellow marks postcodes, in which positive and negative farms were found. The map was created with Excel.

Sock swabs were collected from different regions in Switzerland as shown in Figure 2. Most samples were collected from areas with a high pig density (https://www.atlas.bfs.admin.ch/maps/13/de/16084_5892_5872_4801/25066.html; accessed on 26 September 2021). Positive and negative farms were evenly distributed without any clustering of positive farms.

Overall, 81 of the 138 tested farms were HEV positive in the RT-qPCR (58.8%). Since fattening pigs are the main shedders of HEV [17], we specifically focused on this age group (Table 2). The majority of positive samples (58.5%) were also detected in fattening pigs. Samples from all stages of the fattening phase showed positive results. From the four fattening farms that were sampled in October 2020 and again in May 2021, three farms were positive in October, but only one of those farms was still positive in May. The other age groups had a lower prevalence than the fattening pigs, however, due to the small sample size in these age groups, the results should be handled with care. The three positive samples from mixed age groups included a sample from pigs aged 6 weeks–5.5 months, a sow with 3-week-old piglets and a sow with 2-week-old piglets and 2-month-old pigs. On three farms, individual samples from the different age groups and in addition a mixed sample (one-for-all), where the same sock swab was used to sample all age groups, were collected. In two of the three cases the negative one-for-all sock swab also reflected the negative results of the individual swabs from the different age groups. However, in the third farm the one-for-all sample was negative, even though two of the sock swabs from the individual age groups were positive (Table 3). The mean Ct values of the different age groups were comparable, apart from the single sample from a pig older than 6 months, which was only very weakly positive.

### 3.2. Phylogenetic Analysis

**Figure 3:**
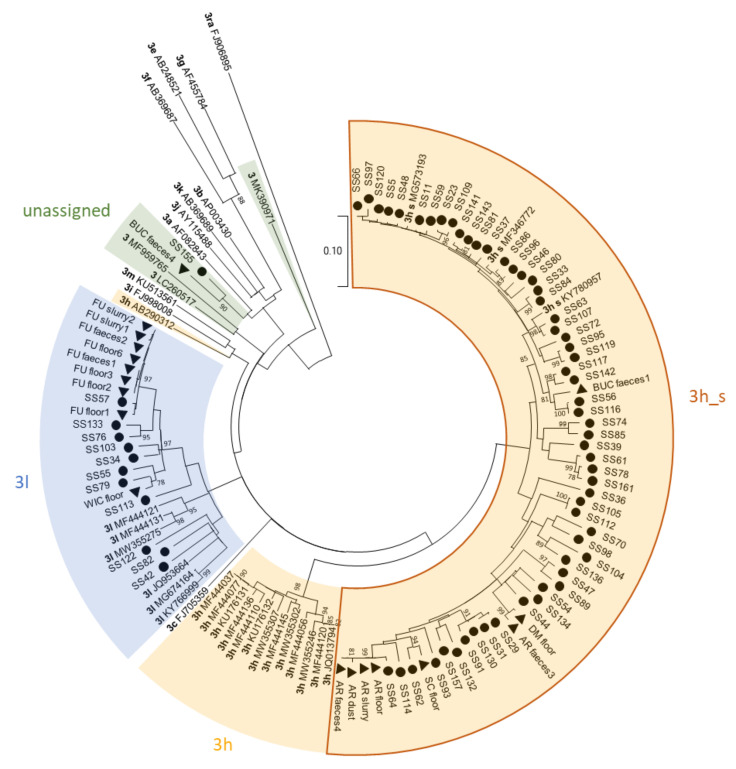
Phylogenetic tree based on partial HEV ORF2 sequences with a length of 493 nt. The tree was created with MEGA X using the Maximum Likelihood method and Tamura-Nei model with 1000 bootstraps. Bootstrap values ≥ 75 are indicated. The analysis included 123 sequences, including 37 reference sequences retrieved from the NCBI GenBank. Reference sequences are labelled with their subtype according to Smith et al. [30] and Nicot et al. [26] in bold and their accession number. Reference sequences from Swiss strains within subtype 3h are labelled with “3h_s”. All strains sequenced in this study are marked with black triangles (▲) for the different sample types and with black dots (●) for the sock swabs. After the triangle the abbreviation of the farm and the sample type are indicated. Identical sequences are collapsed.

Samples from all different sample types could be sequenced successfully. Overall, 96 out of the 115 samples (83.5%) that were submitted to the typing PCR could be sequenced successfully (Table 4). This success rate was similar for all different sample types for which at least five samples were analysed. All partial ORF2 sequences belong to genotype 3. Most samples were assigned to subtype 3h (71 samples), 23 samples to 3l, and 2 samples could not be assigned to any subtype. Furthermore, one individual faecal sample and three environmental samples contained sequences from several different HEV strains, which caused superimposed electropherograms and ambiguities in the sequence. Within subtype 3h, the Swiss strains all cluster together (Figure 3), whereas Swiss sequences in subtype 3l are mixed with sequences from other countries and do not form a separate cluster. The two unassigned sequences clustered most closely with subtypes 3a, 3b, 3j and 3k. For our analysis we included subtype 3j within subtype 3a, as suggested by Nicot et al. [26]. The mean of the pairwise identities for sample BUC_faeces4 compared to subtypes 3a and 3b was 87.8 and 88.6%, respectively (Figure S2). For sample SS155 the means were 88.4% for subtype 3a and 89.5% for subtype 3b. For both unassigned sequences, statistical analysis showed significantly higher pairwise identities with subtype 3b compared to subtype 3a (*p* = 0.004 for BUC_faeces4; *p* = 0.00006 for SS155). Thus, the sequences were most closely related to subtype 3b.

On the three farms from which different sample types were sequenced, different strains were found within one farm (Table 5). On farm AR sequence A was found in all environmental samples while a different sequence was found in one of the faecal samples (B) which only had a pairwise identity of 92.5–93.1% to sequence A. On farm BUC there were also two different sequences detected in the individual faecal samples (C and D) which had a pairwise identity of 87%. In the floor swab and slurry sample from that farm a mixture of these two sequences was present, as shown by in silico separation of the superimposed electropherograms. Furthermore, sequence C from that farm was identical with the sequence from a floor swab from another fattening farm (LE). However, the pigs from these two farms originated from the same farrow and rearing farm. On farm FU (rearing and finishing farm) several pens were sampled within a time span of 4–5 months. On that farm there was one individual faecal sample (F) with a coinfection and a pairwise identity of only 85.8–86.4% to sequence E.

There were three sock swabs from different fattening farms with identical sequences. However, we do not know if there is any connection between these three farms or not. Otherwise, all sequences from sock swabs differed from each other, though in some cases only by one nucleotide. Unfortunately, from the farm that tested HEV positive twice, first in October 2020 and again May 2021, only one of the samples was successfully sequenced.

### 3.3. NGS

We compared three different RNA extraction methods, two using a commercial kit based on silica columns and one using chemical separation of RNA, for full-length sequencing from sock swabs by NGS. The TRIzol extraction worked best for both samples (Table 6). The HEV genome of one sample (SS48) was almost fully covered; only 34 nucleotides at the 5’ end and the poly-A tail at the 3’ end was missing compared to full-length HEV 3h_s references, resulting in the complete ORF 2 and ORF 3 while the ORF 1 lacked 9 nucleotides at the 5’end. This sequence was also identical with its corresponding Sanger sequence. With a coverage of 65.5%, the other sample (SS66) was only partially covered with the TRIzol method, with gaps throughout the genome. For the two methods that used the RNA kit the results differed, depending on the sample. In one case the method using BME resulted in better coverage, in the other case the method using DTT. After the RNA extraction an RT-qPCR was carried out. The corresponding Ct values are indicated in Table 6. The Ct values between the different methods varied only slightly.

**Figure 4:**
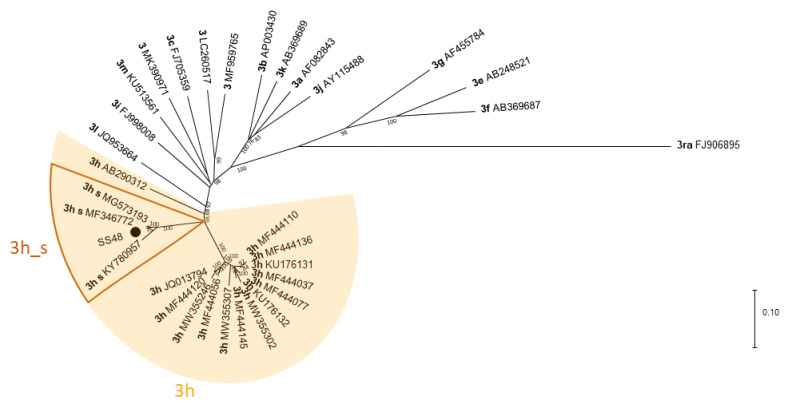
Phylogenetic tree based on HEV (almost) full-length sequences. The tree was constructed with MEGA X using the Maximum Likelihood method and General Time Reversible model with 1000 bootstraps. Bootstrap values ≥ 75 are indicated. The analysis involved 33 sequences, including 32 reference sequences received from the NCBI GenBank. Reference sequences are labelled with their subtype according to Smith et al. [30] and Nicot et al. [26] in bold and with their accession number. Reference sequences from Swiss strains within subtype 3h are labelled with “3h_s”. (●) HEV strain sequenced in this study (accession number MZ923557).

As for the partial ORF2 sequences, the full-length Swiss sequences, including SS48 (MZ923557) formed a separate cluster within subtype 3h (Figure 4).

### 3.4. Questionnaire

The farm size varied from 20–1920 animals per farm, with a mean of 340 pigs (Table S2). Samples were collected from 16 farrowing farms (two also with gilt production and one with fattening pigs), 95 fattening farms (one also with gilt production), 2 rearing and finishing farms, 3 gilt producing farms, and 22 farrow-to-finish farms. Most samples were collected from healthy animals, but 18 farms (occasionally) had problems with diarrhoea, 2 farms with coughing and 2 farms with tail-biting. At the time of sampling only one farm used a treatment, namely antibiotics.

## 4. Discussion

Domestic pigs form the main reservoir of the zoonotic HEV genotype 3. However, more information about origins and pathways of transmission in pig herds is needed to establish measures that prevent further transmissions. An important basis for that is a sequence database which can be used for molecular epidemiology and tracing. To detect HEV on pig farms, so far mainly blood or individual faecal samples were used. However, the collection of these sample types is invasive, time- and labour-consuming and expensive. Therefore, the aim of this study was to establish a practical, non-invasive and cost-efficient screening tool which should also provide samples that are suitable for sequencing. Thus far, only a few non-invasive HEV farm-screening tools are described. One of them is testing slurry; however, in a previous study no correlation between the presence of HEV in individual faecal samples and manure ditches was found [13]. Another method described is the collection of several individual stool samples directly from the floor [42]. This method had very high sensitivity and specificity, but it can require testing of up to 40 samples per farm. Therefore, we were looking for an easier and cost-efficient way and tested different environmental samples, including floor swabs, dust swabs and slurry samples, and compared the results to individual faecal samples. From the different environmental samples that were tested, the floor swabs and slurry samples provided the best results with almost the same number of positive farms detected. However, while all floor swabs were negative after cleaning the pens, more slurry pits were positive. Slurry pits are not cleaned that regularly, which leads to a longer persistence of the virus in slurry. Furthermore, there seems to be a time delay between the pigs shedding the virus and the detection of the virus in slurry. Thus, floor swabs seem to be more reliable to determine the current presence of HEV on pig farms. Furthermore, a reason why more slurry samples were positive could be that the slurry was a collective sample from several pens or even the entire farm, while the floor swabs were only taken from a single pen. Besides, slurry pits are not always easily accessible, which makes their collection more difficult and time-consuming. For these reasons, floor samples in the form of sock swabs were chosen for the screening. Moreover, sock swabs were found to be a reliable diagnostic tool for the detection of other intestinal pathogens in pig herds and are routinely used for *Salmonella* surveillance in poultry [43,44]. However, none of the environmental samples we tested detected all positive farms. Thus, screening herds using environmental samples will probably underestimate the true prevalence. Furthermore, a weakness of the different sample type testing was that individual faecal samples were not taken from all pigs within one pen, which likely led to an underestimate of the number of farms with positive individual faecal samples.

For the sock swab, screening samples from German and French speaking regions in Switzerland—but mostly from regions with a high pig density—were taken. We did not find a clustering of positive cases, which agrees with the findings of Burri et al. [22] of no correlation between pig farm density and positive sample density. In September 2021, shortly before the submission of this paper, we also received a sock swab from Ticino, the Italian speaking part of Switzerland south of the alps, which has a low pig farm density. The sample was HEV negative, but due to the short notice the result is not included in the results section. In our sock swab screening, 58.8% of the farms were HEV positive. This viral prevalence is comparable to the seroprevalence found in 2011 in Swiss pigs [22,23]. Thus, sock swabs seem to be a suitable screening tool to detect HEV in most positive pig herds and it seems that the prevalence has not changed much over the past few years. However, a detection of all positive HEV herds cannot be guaranteed. For example, on one farm, a one-for-all sock swab sample was negative, even though two samples from individual age groups were positive. Thus, it might be necessary to take several samples per farm, especially if different age groups are sampled. More studies are needed to determine the exact number of samples that should be taken, as this might depend on the size of the farm and farming practices or production types. From our study we cannot make any conclusion about these factors, due to relatively small sample size and non-representative sampling. Yet, it seems logical to screen fattening farms, since fattening pigs are the main shedders of HEV as proposed by Salines et al. [17] and confirmed in our study (58.5% prevalence). However, there were also samples collected from other age groups, and positive samples were found in all of them, including two samples from sows with their piglets. This confirmed the results of other studies that found HEV in all age groups, from piglets as young as 3 weeks of age up to breeding sows [13,18,21]. As an additional benefit, sock swabs could also be used for the screening of enteric pathogens in pig herds such as coronaviruses or rotaviruses.

All different sample types in our study could be sequenced successfully. On two fattening farms whose pigs originated from the same farrow and rearing farm, identical sequences were found. Thus, it is likely that the virus was spread to new farms with the introduction of infected pigs. This could also explain why different sequences were present within one farm, since pigs from different farms may be mixed. The presence of different HEV strains in a single farm and the same HEV strain in different farms was noticed before, also suggesting a common source of infection [18]. On some farms where different strains were present, a mixture of these sequences was found in the environmental samples. Consequently, not all environmental samples could be sequenced successfully using the Sanger method, which is a disadvantage of using mixed samples. However, in rare cases this problem can also arise in individual samples, as shown by a co-infection with several HEV-3 strains in one of the individual faecal samples, a finding that was reported before in Brazil [45]. Within one farm, there were also sequences that differed slightly (up to 1.4%) from each other. Thus, it may be possible that the virus evolved on the farm level since mutations occur frequently in RNA viruses and the existence of HEV quasispecies was reported [46]. Therefore, it would be interesting to retest positive farms regularly, to monitor how fast the virus evolves in the natural reservoir.

The sock swabs also proved to be suitable for sequencing, since over 80% of the RT-qPCR positive sock swabs were successfully sequenced with the Sanger method. There were three farms with an identical sequence, but the origin of the pigs from these farms was unknown. Otherwise, all strains sequenced from the sock swabs were different from each other. Thus, in most cases it would be possible to trace back an infection to a specific farm. The potential of tracing back infections to a farm level was already shown in a Swedish study in which unique farm-specific strains were found that remained on the same farm over a two-year period [47]. However, this study included only farrow-to-finish farms while our study included also farms which received pigs from other farms, which can complicate molecular tracing.

All sequences found in this study were assigned to genotype 3. Most of them belonged to subtype 3h. This subtype was already found to be the most prevalent one in humans in Switzerland [31]. Within subtype 3h, the Swiss sequences formed their own cluster, formerly known as 3s(p). A reason for this could be the relatively isolated Swiss pig population. There is very little import and export of living pigs, and not much pork is imported or exported (https://www.proviande.ch/de/der-fleischmarkt-in-zahlen; accessed on 1 September 2021). Interestingly, a similar Swiss-specific cluster was detected in atypical porcine pestivirus (APPV) strains isolated in Switzerland, supporting an exclusive virus circulation of certain strains limited to Switzerland [48]. However, there were also other HEV subtypes detected in Swiss pigs. Some of the sequences belonged to subtype 3l, formerly referred to as 3o(p). This subtype was also found in Swiss patients [31], but also in patients and pigs from other countries such as France and Italy [30]. Furthermore, there were two sequences which could not be assigned to any subtype. Interestingly, those two subtypes were most closely related to subtype 3b, which was mostly found in Asia so far, though it was also detected in humans and animals in Europe before [26,49,50,51]. Therefore, the introduction of new subtypes to Switzerland, for example through the import of pigs, cannot be ruled out. However, for classification of these two samples full-length sequencing would be required. Due to the limited length, the partial ORF2 segment we used for Sanger sequencing may not always be representative for the full-length genome. Furthermore, genetic resolution was shown to be lower when sequencing short fragments [52]. Subtyping is not always reliable with shorter fragments, whereas genotyping is still possible with fragments as short as 200 nt [52]. Yet, the method described by Boxman et al. [38], which we used in this study, proved to deliver good results and is widely used in different countries [53], thus allowing international comparison of the sequences.

We did attempt full-length sequencing with two sock swabs using three different methods to see which one is most suitable for sequencing HEV. Even though both samples had comparable Ct values (31.0 and 31.8), the coverage varied substantially between these two samples, which may be due to differences in the sample quality, i.e., RNA integrity. However, the sample with the lower coverage (SS66) had slightly higher Ct values after extraction with the different methods. Therefore, better NGS results might be received from samples with lower Ct values. For both samples, the best coverage and most reads were achieved using the TRIzol extraction. With this method, one sample (SS48) was almost fully covered. The sequence was identical with its corresponding Sanger sequence and was also assigned to subtype 3h. Thus, sock swab samples can also be used for NGS, allowing longer sequences to be generated, thereby enhancing molecular tracing and HEV subtyping.

## 5. Conclusions

In our pilot study, sock swabs were established as an easy-to-use, non-invasive and cost-efficient screening tool for HEV in pig herds and proved to be suitable for Sanger and next-generation sequencing. In a first pilot study using this screening tool we confirmed a high HEV prevalence in Swiss pig herds and showed subtype 3h to be most prevalent. However, further studies are needed to refine the sampling and determine the exact number of samples needed per farm.

## Figures and Tables

**Table 1:** Summary of the hepatitis E virus (HEV) RT-qPCR results from the different sample types from all fattening farms and from the rearing and finishing farm.

Number of Farms	Environmental Samples			Individual Faecal Samples
	Floor Swab	Slurry	Dust Swab	
6	+ ^1^	+	+	+
3	+	+	− ^2^	+
1	+	+	−	−
2	−	+	−	−
2	−	−	−	+
1	−	−	−	−
Mean Ct value	36.4	37.8	37.5	37.5
Range of the Ct	32.7–39.9	32.4–41.1	35.0–39.6	27.5–42.6

^1^ + positive; ^2^ − negative.

**Table 2:** Results of the HEV RT-qPCR from the sock swab screening.

Age of Pigs Tested	No. of Total Samples	Positive Samples		Mean Ct Value	Range of the Ct Value
		No.	%		
4–10 weeks	12	3	25.0	33.8	28.5–40.6
>10 weeks–6 months	130	76	58.5	34.8	25.9–43.2
>6 months	3	1	33.3	40.3	
Mixed age groups	8	3	37.5	34.6	29.0–37.5
Total	153	83	54.2	34.8	25.9–43.2

**Table 3:** Summary of the RT-qPCR results from sock swabs from three farms that had a one-for-all as well as individual sock swabs sampled from the different age groups.

Farm	4–10 Weeks	>10 Weeks–6 Months	>6 Months	One-for-All Sample
A	+	−	+	−
B	−	−	Not sampled	−
C	−	−	–	−

**Table 4:** HEV subtyping results from the Sanger sequencing of the 493 nt long ORF2 region. Subtypes were assigned with help of the Hepatitis E Virus Genotyping Tool and the phylogenetic tree we constructed.

Sample Material	No. of Samples Submitted to Typing PCR	No. of Typing PCR Positive Samples	No. of Successfully Sequenced Samples	Subtype 3h	Subtype 3l	Unassigned Genotype 3
Floor swabs	13	12	11	4	7	-
Slurry	5	5	4	1	3	-
Dust swabs	2	1	1	1	-	-
Individual faeces	12	11	10	7	2	1
Sock swabs	83	71	70	58	11	1
Total	115	100	96	71	23	2

**Table 5:** Different sequences found on four farms from which different sample types were tested.

Sample Type	AR	BUC	LE	FU
Floor swab	A	C + D	C	E
Slurry	A	C + D	− ^1^	E
Dust swab	A	−	−	−
Individual faeces	3 × A, 1 × B	3 × C, 1 × D	−	2 × E, 1 × F

^1^ − samples were either not submitted to the typing PCR or were negative in the typing PCR.

**Table 6:** Summary of the results from the next-generation sequencing (NGS) of HEV from two sock swabs.

Sample	Method	Ct Value	HEV Coverage (%)	Reads ^4^
SS48	TRIzol	31.5	99.4 ^1^	3259
SS48	RNA kit with BME	31.0	50.6 ^2^	43
SS48	RNA kit with DTT	30.5	60.6 ^1^	65
SS66	TRIzol	34.1	65.5 ^1^	105
SS66	RNA kit with BME	32.0	18.6 ^1^	10
SS66	RNA kit with DTT	32.0	3.0 ^3^	33

^1^ Genome coverage in percentage relative to reference strain MG573193; ^2^ relative to reference MF346772; ^3^ relative to reference MF444119; ^4^ number of total reads detected regarding the best reference strain, as mentioned for the coverage.

## Data Availability

The sequences generated in this study are available in NCBI GenBank) under the accession numbers MZ923557–MZ923643. NGS raw data are deposited in NCBI Sequence Read Archive (SRA) as BioProject PRJNA767214 (BioSamples SAMN21894383-SAMN21894388). Supplementary Materials showing detailed results of this study are available online.

## Supplementary Materials

The following are available online at https://www.mdpi.com/article/10.3390/ani11113050/s1, Figure S1: Picture of a sock swab fixed to a boot to sample the pig pen floor; Figure S2: Pairwise identities, Table S1: Different sample types: farms; Table S2: Sock swabs.
