# Supplementary material for: Screening of Swiss Pig Herds for Hepatitis E Virus: A Pilot Study
Source: Animals (Basel). 2021 Oct 25;11(11):3050. doi: 10.3390/ani11113050 (PMC8614339; doi:10.3390/ani11113050)
Supplement: Supplementary file 1 [file animals-11-03050-s001.zip › animals-1424045-supplementary/Figure_S2_Pairwise_identities.pdf]

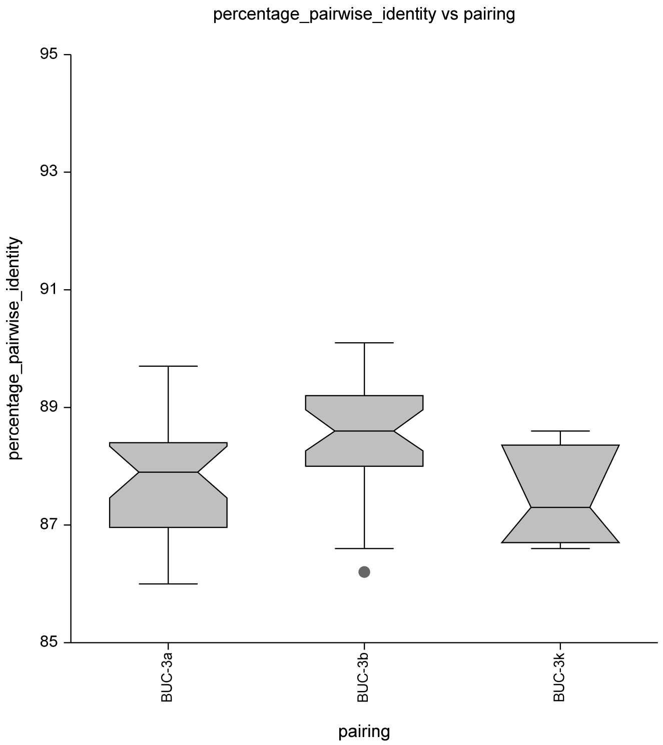

(a)

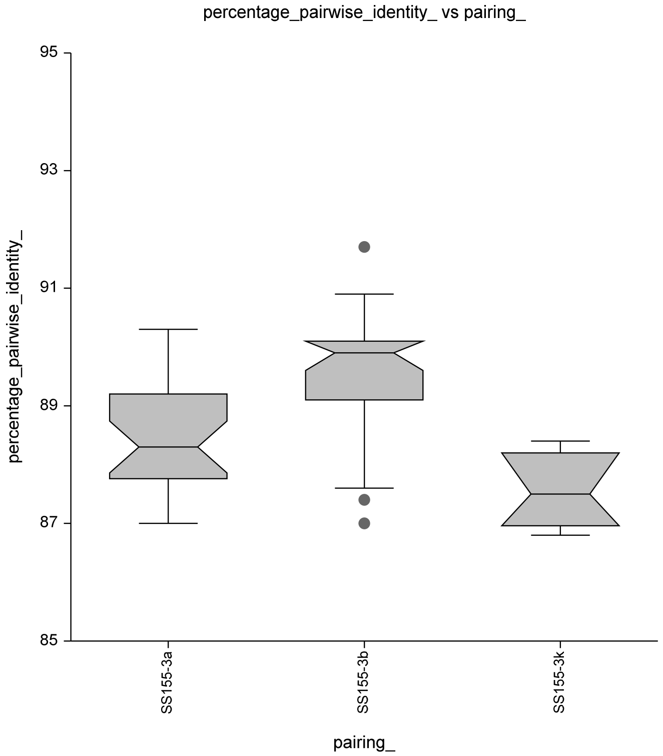

(b)

Figure S2. Pairwise identities of the two unassigned sequences BUC_faeces4 (a) and SS155 (b) compared to all members of subtypes 3a (n=26), 3b (n=29) and 3k (n=4) as assigned by Nicot et al (2020)[26].
