# Supplementary figures and images for: Screening of Swiss Pig Herds for Hepatitis E Virus: A Pilot Study
Source: Animals (Basel). 2021 Oct 25;11(11):3050. doi: 10.3390/ani11113050 (PMC8614339; doi:10.3390/ani11113050)

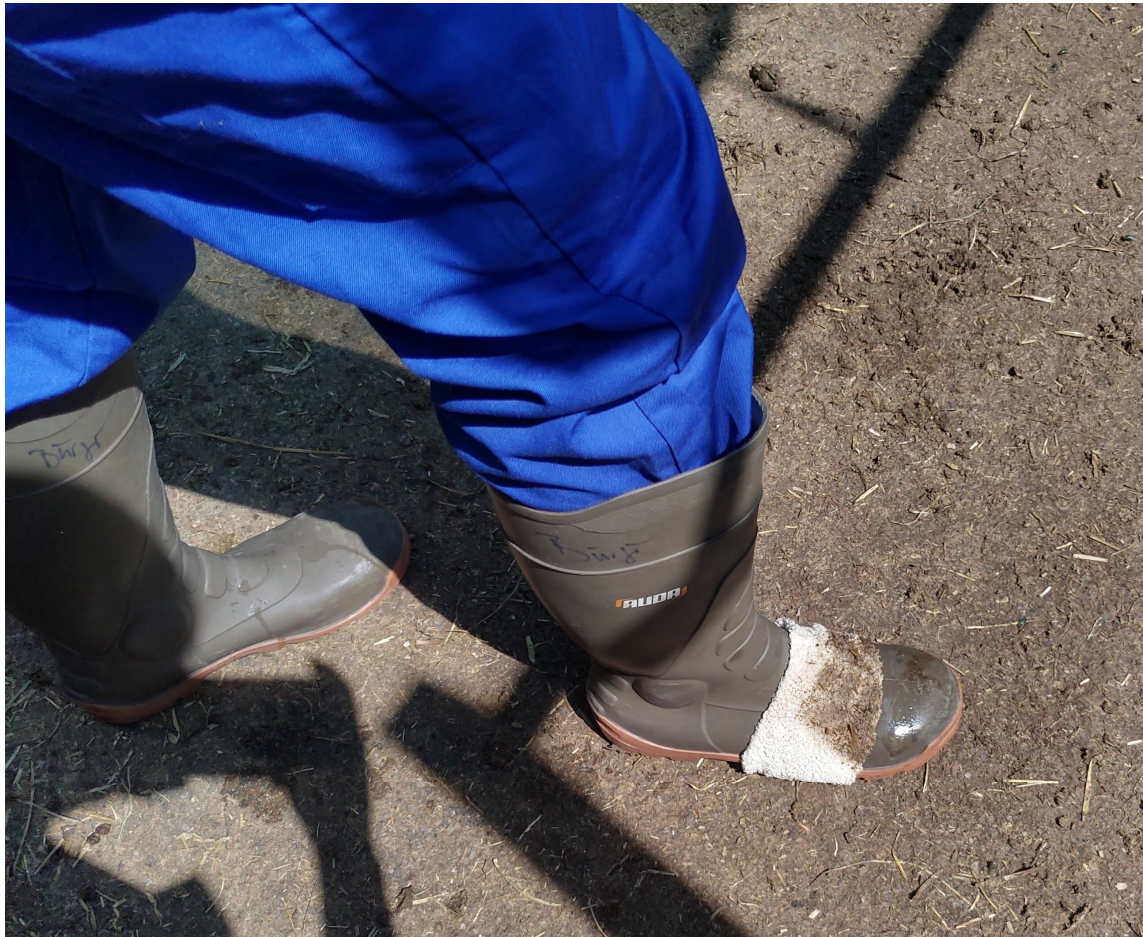

**Figure S1.** Picture of a moistened sock swab affixed to a boot to sample the pig pen floor.

Supplement: Supplementary file 1 [file animals-11-03050-s001.zip › animals-1424045-supplementary/Figure_S1_sock-swab-picture.pdf]
